# Risk perceptions and behaviors of actors in the wild animal value chain in Kinshasa, Democratic Republic of Congo

**DOI:** 10.1371/journal.pone.0261601

**Published:** 2022-02-16

**Authors:** Ashley Lucas, Charles Kumakamba, Karen Saylors, Erby Obel, Reggiani Kamenga, Maria Makuwa, Catherine Clary, Guy Miningue, David J. McIver, Christian E. Lange, Placide Mbala Kingebeni, Jean J. Muyembe-Tamfum

**Affiliations:** 1 Labyrinth Global Health, Saint Petersburg, FL, United States of America; 2 Metabiota Inc., San Francisco, CA, United States of America; 3 Metabiota Inc., Kinshasa, Democratic Republic of the Congo; 4 Metabiota Inc., Nanaimo, BC, Canada; 5 UCSF, San Francisco, CA, United States of America; 6 Institut National de Recherche Biomédicale, Kinshasa, Democratic Republic of the Congo; CDC, UNITED STATES

## Abstract

In the Democratic Republic of Congo (DRC) which contains the greatest area of the second largest rainforest on Earth, people have long been connected to the forest for subsistence and livelihood from wild animals and bushmeat. This qualitative study sought to characterize the bushmeat movement—from hunting wild animals to market sale—and the roles of participants in the animal value chain, as well as their beliefs surrounding zoonotic disease and occupational risk. Actors in in eight bushmeat markets and two ports in Kinshasa, DRC completed semi-structured interviews between 2016 and 2018 in which they expressed belief in transmission of illness from domestic animals to humans, but not from wild animals to humans. Wild animals were viewed as pure and natural, in contrast to domestic animals which were considered tainted by human interference. Participants reported cutting themselves during the process of butchering yet did not consider butchering bushmeat to be a risky activity. Instead, they adopted safety practices learned over time from butchering experts and taught themselves how to butcher in a fashion that reduced the frequency of cutting. In general, butcherers rejected the idea of personal protective equipment use. Port markets were identified as important access points for meat coming from the Congo river and plane transport was identified as important for fresh and live meat coming from Équateur province. Most participants reported having heard about Ebola, but their mistrust in government messaging privileged a word-of-mouth story of witchcraft to be propagated about Ebola’s origins. It is critical to better understand how public health messaging about outbreaks can successfully reach high risk communities, and to develop creative risk mitigation strategies for populations in regular contact with animal blood and body fluids. In this paper, we offer suggestions for formal and informal trusted channels through which health messages surrounding zoonotic risk could be conveyed to high-risk populations in Kinshasa.

## Introduction

Bushmeat is a term used to describe meat obtained from free ranging wild animals, primarily in the forests and savannahs of Africa and Asia. The economic, social, and subsistence role of bushmeat in Central Africa has been well documented [1, 2] and humans have been hunting in Central African forests for generations.

It has been estimated that one million metric tons of bushmeat are consumed each year in the Congo Basin. Although meat consumption in the Congo Basin is equivalent to that of Europe, about 80% of this meat is derived from wildlife, rather than from domestic animals [3]. Bushmeat provides subsistence and protein to supplement diets, and potential income to those that hunt and sell. Hunting, butchering, and selling practices and traditions are passed down through families. From suppliers to consumers, rural to urban populations, bushmeat is an essential staple of life in Central Africa [2, 4, 5].

Bushmeat is also recognized as a source of emerging pathogens with epidemic and pandemic potential of global importance and a major global public health threat [6–8]. With the second largest rainforest on earth, the Congo Basin is considered a potential hot spot for such emergence events, as it contains high species diversity and experiences hunting, deforestation, rapid urbanization and population growth, which all serve to bring wild animals and humans into close contact [3]. A number of historic spillover events have been linked to wild animals in Central Africa, such as the emergence or re-emergence of HIV-1, Ebola virus and Monkeypox virus [9–13], and new pathogens with spillover potential are frequently being discovered in key species such as bats, non-human primates, and rodents [14, 15].

Kinshasa is the Democratic Republic of the Congo’s (DRC) largest city and home of close to 12 million people (2017 est; Demographia, 2019). It lies on the southern bank of the Congo River spreading out southward from the shore at Malebo Pool, a widening of the river. It sits directly across the river from Brazzaville, the capital city of the Republic of Congo, and movement between the two countries for trade is frequent. The main crops of the surrounding area are cassava, cane sugar, palm oil, plantains, corn, peanuts and beans. The population of the city is young, with the majority of the population in their twenties or younger. Population growth since the country’s independence in 1960 has been driven by a rural exodus, with many people leaving the political instability, conflict, and economic decline of rural areas in order to migrate to Kinshasa [16].

The impact of hunting and bushmeat trade in DRC (particularly primate species such as gorillas, chimpanzees, and bonobos) is well-documented [17, 18], as is the impact of hunting/poaching in protected areas [19]. However, there is no literature focused specifically on the bushmeat trade in the capital city of Kinshasa. Kinshasa is known to have a large and dynamic bushmeat trade, with formal and informal markets scattered across the city. These markets sell everything from caterpillars to reptiles, to bats, to non-human primates, ungulates and rodents.

No outbreaks or known zoonotic pathogens of consequence have emerged from bushmeat markets of Kinshasa and human outbreaks of Ebola and Monkeypox have frequently been linked to rural areas with poor health infrastructure [20]. However, rapid urbanization of Central Africa, where 50% of the population is expected to reside in urban areas by 2030 [11] necessitates a closer look at the dynamics of bushmeat trade in large urban hubs. Mbandaka, the Capital city of Equateur province, with a population of approximately 1.2 Million people, has been the largest city in DRC to witness an outbreak of Ebola, though spillover event[s] were not linked directly to this urban area [21]. Demand for bushmeat by many urban consumers has created a substantial market in Kinshasa [22] which, in turn, may cause elevated risk of pathogen spillover to the population. To reduce the risk of emergence of new zoonotic pathogens of epidemic and pandemic potential, public health officials must understand the reasons why people eat bushmeat, behaviors of participants within the bushmeat trade, as well as the workings of the bushmeat value chain itself. Beliefs, practices and behaviors associated with wild animal hunting, transport and trade can help public health officials can develop locally appropriate mitigation for high-risk sub-populations in frequent contact with animal body fluids, a known risk factor for virus spillover [23].

## Methods

Zoonotic disease research designed to combat pathogens that could spark future pandemics was conducted through the USAID funded PREDICT-2 project in Kinshasa markets between July 2016 and October 2018. PREDICT-2 worked with select countries to monitor behaviors, practices and conditions associated with viral evolution, spillover, amplification and spread, as well as improve surveillance through predictive modeling and begin developing risk-mitigation strategies to reduce zoonotic virus risk [24]. We adopted a One Health Approach which recognizes the advantage of looking at a complex health and/or environmental problem from the multidisciplinary perspective of environmental, animal, and human health [25]. We used a qualitative approach to One Health for this study as described by Saylors et al., with observational research, semi-structured interviews and focus groups. This methodology was designed to capture different facets of zoonotic disease emergence, including socio-cultural behaviors of individuals living and working at the human, animal and environmental interfaces that may be influential in pathogen spillover, amplification and spread not captured in traditional outbreak disease models [26]. Specifically, we aimed to gain a deeper understanding of 1) how and from where bushmeat (smoked, fresh, live) arrives to Kinshasa markets 2) The beliefs, perceptions, and behaviors of actors in the bushmeat trade about wild animals and their handling, sale, consumption, and 3) descriptively and qualitatively assess the risks of zoonotic transmission in bushmeat value chain actors in Kinshasa markets. With this understanding we aim to provide an overview of the flow of bushmeat to the capital Kinshasa, identify beliefs and practices of market workers surrounding zoonotic disease and working with wild animals, and recommend strategies to reduce exposure and improve education surrounding zoonotic disease in bushmeat workers in markets across the city of Kinshasa, DRC.

### Observational research, interviews, and focus groups

**Fig 1:**
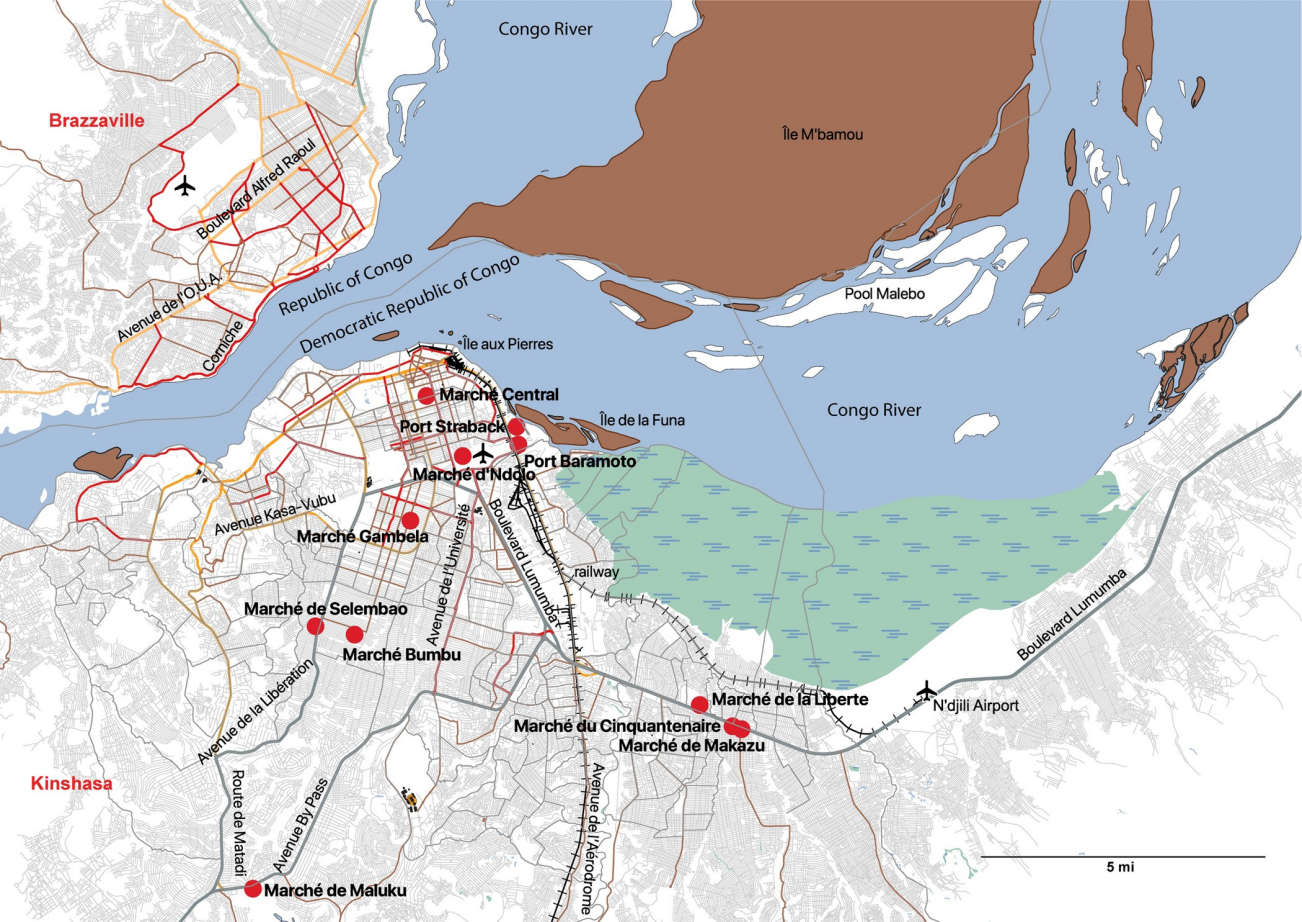
Map of Kinshasa study sites. Included are bushmeat markets where sampling occurred, main road arteries, location of the International airport, the Congo river and border designation between Democratic Republic of Congo and Republic of Congo. Base map and data from OpenStreetMap and OpenStreetMap Foundation, which is made available under the Open Database License.

Observational research was conducted at markets in Kinshasa, where semi-structured interviews and focus groups took place with participants. Researchers observed market layout (bushmeat location in relation to other merchandise and toilets/garbage), cleanliness (garbage containers, sewage, hand washing, soap availability, cleaning stall frequency), water accessibility (approximate distance to clean water supply), wild meat selection, meat storage (freezer, cold boxes, none), price, and meat state (fresh/recently slaughtered, live, smoked). Semi-structured interviews were conducted with bushmeat actors at markets throughout Kinshasa. We selected markets throughout the city with varied wild animal species and two port markets adjacent to the Congo River. In total we conducted observational research, 59 semi-structured interviews, and two focus groups at eight urban markets and two port markets between July 2016 and January 2018: Makazu, Gambela, Cinquantenaire, N’Dolo, Liberté, Selembao, Central, Bumbu, Port Strabak, and Baramoto (Fig 1). We used a purposive sampling method, with the goal of interviewing all bushmeat vendors at a select market. We additionally used snowball sampling to recruit other participants in the animal value chain (such as middlemen and hunters) based on information from market vendors. Participants in both interviews and focus groups were required to have indirect or direct contact with live animals. Direct contact includes raising, hunting, selling, trading or purchasing live, freshly killed or smoked animals/animal meat. Indirect animal contact includes animals living in or entering dwellings, buildings or gardens/crops (e.g., bat roosts along roofs, rats or other animals invading stored food or crops). Because participants were a targeted sub-population, they were not a representative sample of the general population. Interview and focus group guides were developed with the PREDICT 2 consortium and intended to be broad enough to cover relevant topics in three continents and 17 countries, therefore not all topics were covered in every country’s study design. The consortium-wide guide covered the following topics: human movement, socioeconomics, biosecurity in human environments, illness, medical treatment, and the nature and frequency of human-animal contact. Semi structured interviews ensured all five dimensions of the interview guide were addressed, yet also allowed interviewers flexibility in focusing on certain areas and further exploring important topics within each domain that arose with an individual during the interview. The focus of interviews and focus groups in DRC markets (selected based on pilot data and data from PREDICT-1) were on the domains of human-animal contact, medical care and treatment, Ebola, and biosecurity. Focus group participants completed a mapping exercise, which involved naming all the animals living in the area where focus group participants worked, completing a visual map of mammalian and avian species. Furthermore, researcher-maintained interview/focus group notes which detailed observations about participant(s) behavior, demeanor and comfort during the interview or group discussion. Interviews lasted approximately 20–40 minutes and focus groups lasted approximately 45–60 minutes. Interview and focus group guides can be found in S1 and S2 Figs.

Participants provided verbal consent to participate in the study and all were given a study information sheet to keep. All qualitative interviews were recorded using digital audio recorders and were transcribed and translated into French (as needed) by local research scientists fluent in Lingala and French. Participants who did not wish to be recorded were excluded from the study. Francophone scientists then followed a deductive yet flexible coding framework [27], guided by a standardized PREDICT Universal Codebook, whereby codes were organized by themes and sub-themes, which was developed using a One Health Framework and based on previous literature and PREDICT-1 work. In order to avoid a rigid approach whereby previous assumptions were applied to new PREDICT-2 data, new codes were added, old codes deleted and/or supplemented as needed [28] until all major themes emerged. Analysis was performed in the software program Dedoose v8. The qualitative codebook is presented as S3 Fig.

### IMPACT study

Between May 28, 2018 and September 13, 2018, research teams in DRC conducted a sub-study embedded within PREDICT 2, termed the IMPACT study, where species diversity and price were observed regularly at two markets in Kinshasa; Gambela and N’Dolo markets (Fig 1). The total data collection period was over a span of 109 days; 65 days of observation at Gambela market by the research staff and 44 days of no observation; 64 days of observation at Ndolo market and 45 days of no observation. “No observation” days were mostly weekends, with the exception of one Saturday (Aug 25, 2018) where research staff visited Ndolo market and not Gambela market, and the team was unable to be present at the markets on the other no observation days due to other commitments. Research staff visited both markets on weekday observation days. Price was recorded by observing transactions between merchants and buyers and asking vendors the price range. Recording focused on the three key taxa of the PREDICT 2 study: non-human primates (NHP), rodents and bats, but also included other taxa including carnivores, ungulates, reptiles and pangolins. Permission was obtained from both market managers and vendors prior to observation.

### Compliance with ethical standards

PREDICT partner Metabiota obtained Institutional Review Board (IRB) permissions from the Western Institutional Review Board for exemption of the qualitative protocol, which was approved on April 6, 2015 and approved by the Kinshasa School of Public Health in April 25th, 2015. The qualitative protocol was renewed by the IRB at the University of California, Davis (UCD) on April 7th, 2016 (IRB ID 754490–3) and by the IRB at the Kinshasa School of Public Health on April 28th, 2016 (IRB Approval ESP/CE/035/2016). For observational research and interviews, we also obtained administrative permissions from market authorities and explained the study goals to market managers prior to speaking with vendors and recording observations.

## Results

Individuals who participated in semi-structured interviews were all adult workers in the wild animal value chain. Twenty-five interviews and one focus group were conducted with participants that identified exclusively as vendors at Kinshasa markets, while the other interview participants performed dual roles such as hunter/transporter and vendor/butcher. Poachers self-identified as hunting illegal/banned species versus hunters. Characteristics of interview and focus group participants are presented in Table 1.

**Table 1:** Demographic and livelihood characteristics of interview and focus group participants in Kinshasa, Democratic Republic of Congo.

*Interviews*							
Site	District	Interview Month	Language	Duration (min)	Gender	Number of Interviews	Occupation
Marché Central	Lukunga	March, 2016	French/Lingala	Long– 30	Female—3	4	Bushmeat Vendor—3
				Short—21	Male—1		Bushmeat Vendor/Butcher—1
Port d’Straback	Lukunga	November, 2016	French/Lingala	Long—25	Male—6	6	Hunter/Supplier—4
							Poacher/Supplier—1
				Short– 14			Bushmeat Vendor/Supplier—1
Marché de la Liberté	Tshangu	March—October, 2016	French/Lingala	Long—42	Female—3	14	Bushmeat Vendor/Butcher -10
							Bushmet Vendor/Supplier—1
				Short—15	Male—11		Bushmeat Vendor—3
Marché du Cinquantenaire	Tshangu	March—October, 2016	French/Lingala	Long—32	Female—3	8	Supplier/Intermediary—5
				Short—15	Male—5		Bushmeat Vendor—3
Marché de Makazu	Tshangu	March—October, 2016	French/Lingala	Long—32	Female—6	7	Intermediary—4
				Short—17	Male—1		Bushmeat Vendor—3
Marché de Ndolo	Funa	April—October, 2016	French/Lingala	Long—27	Male—8	8	Bushmeat Vendor—8
				Short—9			
Marché de Bumbu	Funa	April, 2016	French/Lingala	Long—29	Male—2	2	Bushmeat Vendor—2
				Short—20			
Marché de Gambela	Funa	October, 2016	French/Lingala	Long—16	Female—4	4	Bushmeat Vendor—2
							Bushmeat Vendor/Supplier—2
				Short– 12			
Port de Baramoto	Lukunga	November, 2016	French/Lingala	Long—32	Female—1	4	Vendor/Supplier—4
				Short—12	Male—3		
***Focus Groups***						**Number of Particpants**	
Marché de la Liberté	Tshangu	January, 2018	French/Lingala	46	Male	8	Bushmeat Vendors/Butchers
Marché de Ndolo	Funa	January, 2018	French/Lingala	48	Male	8	Bushmeat Vendors

### Bushmeat movement to Kinshasa markets

**Fig 2:**
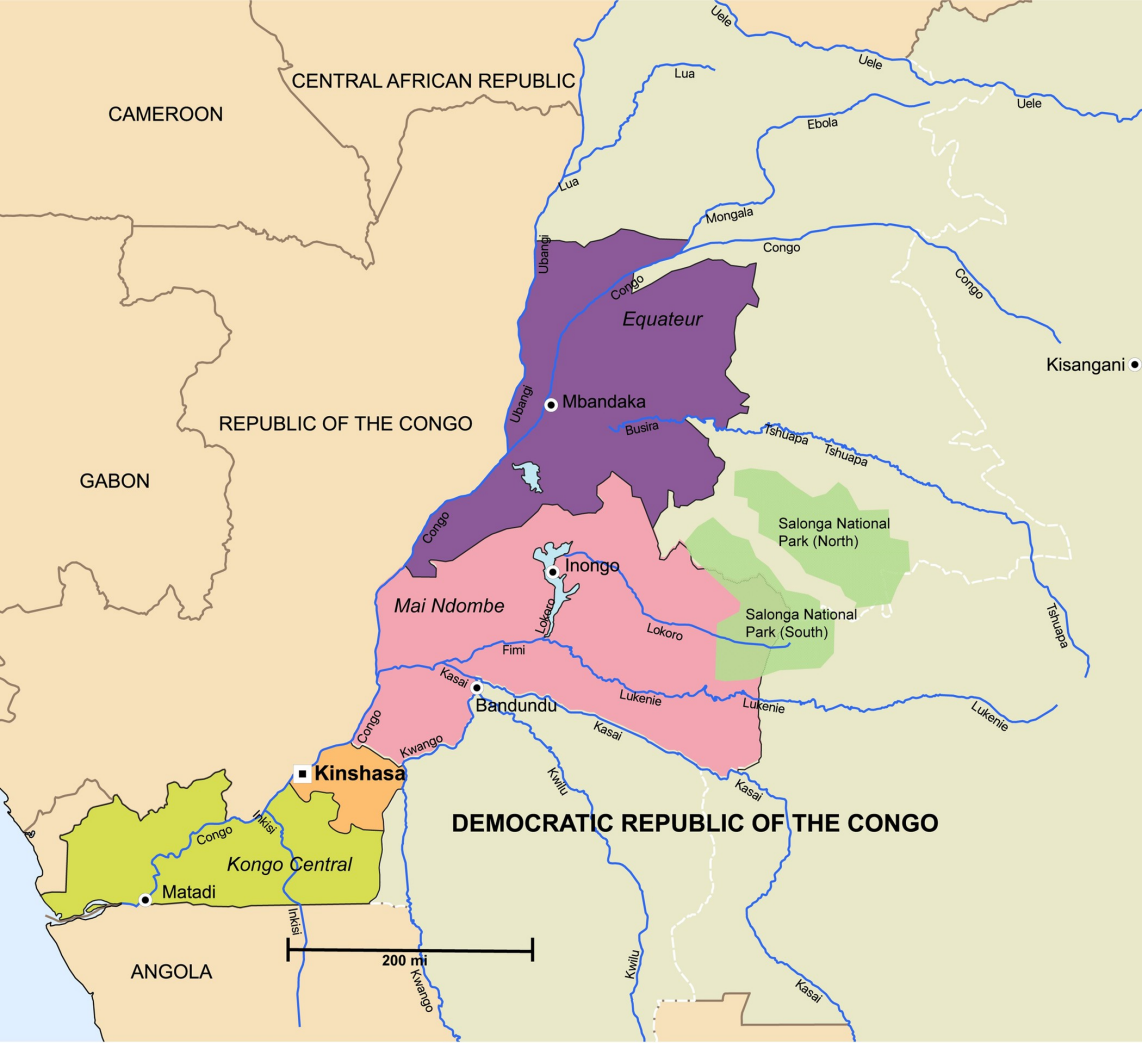
Regional map of the three main provinces for export of bushmeat to Kinshasa markets. Illustration showing participant-indicated provinces where bushmeat is sourced for hunting, transport and sale in Kinshasa bushmeat markets. Kinshasa is both a province and a city, with the former indicated by the white and black square and the latter in orange. Capital cities of each province are indicated by white and black circles and province names are italicized. Former province borders (the six largest were split in 2015) are indicated with a white dashed line.

When asked about where meat comes from, participants mentioned three regions as a source of bushmeat. Kongo Central (formerly Bas Congo) is in Western DRC and borders the Atlantic Ocean as well as the Republic of Congo and the Republic of Angola (Fig 2). From this region, bushmeat is transported to the capital, Kinshasa, by car or by bus in wrapped parcels. Hunters catch animals, immediately freeze the meat and wrap them for transport. There are transport services specifically designed for this purpose, so a hunter or middleman does not always need to physically travel with the meat. Vendors report that meat arrives still frozen after the 24- to 48-hour bus journey. One individual who has been working in the bushmeat trade for over 20 years reported that there is not much meat left in the Kongo Central region (compared with when he began working in the bushmeat trade), and another confirmed that bushmeat does not come from that region as often as it did in years past. Both participants state that in order to get good quality bushmeat in abundant quantities you must source it from farther into the forest, specifically locations in the Équateur province (Fig 2).

Équateur is to the northwest of Kinshasa and borders the Republic of Congo to the West. The Congo River flows through Équateur province down to Kinshasa. The province has witnessed three Ebola outbreaks in the last decade, including the most recent outbreak in 2020. Équateur is home to large areas of tropical forest and several large national parks. Due to the distance from Kinshasa, meat is often transported to the capital by plane through Ndolo airport, which is adjacent to Ndolo market. This meat is often freshly killed (according to participants this means the “blood is still flowing”) and is expensive compared to other modes of transportation and other source locations from which bushmeat is procured. Not many vendors have the capital to source meat from this region, specifically the bushmeat that is transported fresh by plane.

Due to Équateur’s proximity to the Congo River, meat is also transported by boat over a period of weeks or months to Kinshasa. The transported meat can be conserved in freezers and cold boxes that can be rented, on the boat, for preservation during the journey, but most commonly, the meat is smoked prior to transport. Protected species NHP are reported as coming from Equateur more frequently than other provinces. Protected species are sold to wealthy Congolese or exported for sale in Europe.

Mai-Ndombe province is sandwiched between the Équateur and Kinshasa provinces (Fig 2). It is home to forested areas of over 10 million hectares, including part of Salonga National Park (South). From Lake Mai-Ndombe, where the capital city of the Inongo province is located, the Fimi River flows south to the Kasai River, which in turn empties into the Congo River. As a result, boats are the main means of transportation for bushmeat from this region. Vendors reported that meat is also transported by car or bus, but not as frequently.

#### Species diversity and bushmeat pricing

In 65 days of active observation, the team recorded a total of 493 bushmeat species pieces at Gambela and 412 at N’Dolo markets. At Gambela market, researchers recorded 32 primates, 132 rodents and no bats. There were additionally four carnivores, 32 ungulates, 293 reptiles and eight pangolins. At the N’Dolo market researchers recorded 160 primates, 96 rodents, and no bats. Additionally, there were 15 carnivores, 20 ungulates, 22 pangolins and 99 reptiles recorded. A summary of species diversity, pricing and IUCN status can be found in S1–S4 Tables.

Prices remained stable for all species in both markets over the observation time. The highest priced species noted in both markets was antelope with a price between $75 and $180 USD at Gambela market, and $80–120 at N’Dolo market.The lowest was tortoise between $6 and $20 USD at Gambela market and $6 and $40 USD at N’Dolo market. All prices quoted are for the whole animal.

### Interview and focus group themes

#### Disease perception

Many participants reported believing that domestic animals get sick and carry transmissible diseases that can be passed to humans. While interviewees believed that wild animals fall ill themselves, they were unanimous in their point of view that wild animals do not transmit disease to humans. Some zoonotic diseases from domestic animals were named, such as rabies, and others were simply described, such as mass “outbreaks” and death among chickens, sheep and goats. Participants cite a deeply rooted connection to bushmeat that spans not only their lives, but generations, and a spiritual connection to the forest as a source of nourishment and livelihood. Wild animals were described to be “God’s creatures” and “sacred,” “pure” and “natural” by participants. Interviewees believe that domestically raised animals can transmit disease to humans since they “eat some of the same food (as) humans,” and are subject to human management and interference. Wild animals, by contrast, were said to “cure” themselves with certain plants found in the forest when they got ill. Some participants mentioned wild animals knew which leaves to eat to treat their ailment if they fell ill, which were also found in their stomachs when butchered.

“Q: In your opinion can domestic animals transmit illness to animals? A: Chickens. Chickens have periods when we know they fall ill. It’s during the dry season. Over time we know that chickens get sick in the dry season. And as soon as its the wet season, its finished. They only get sick in the dry season.” *(Bushmeat Vendor*, *Female*, *Marché de la Liberté)*.

#### Low perceived risk of contact with animal blood and butchering

Many participants do not consider hunting to be a risky activity, nor were they concerned about regular contact with animal blood or butchering with open wounds in the market. All animal value chain workers that we interviewed had been raised in a culture of hunting, butchering, and eating wild animals. As such, perceived risk for disease transmission is low or non-existent. Subjects answered “yes” to frequent daily contact with animal blood but were unconcerned with this contact and perceived butchering as only slightly dangerous—due to the risk of cuts. When asked if they considered these or other job-related activities dangerous, subjects seemed surprised at the question. To many participants, occasionally cutting themselves while butchering is unavoidable, and the risks are reduced by techniques learned over time from experienced butchers. Vendors in Kinshasa, however, reported being very skilled in this task and cuts were infrequent. The idea of personal protective equipment (PPE) use was discarded as being cumbersome, as gloves were “too slippery” to use while butchering, rendering the risk of cutting oneself higher. In this way the perception of “risk” by participants was different than researchers: participants worried about cutting themselves and having to take time off work.

#### Hand washing

Although the question of whether or not a vendor washed his/her hands was not asked consistently, most participants who were asked reported washing their hands regularly, either after eating, at the end of the day, or in between different tasks at the market. These hand-washing points were considered important for cleanliness, as well as rinsing the hands of any visible blood for other customers. However, considering there is no direct access to water near bushmeat vendors in any of the markets, hand washing at the market would encompass a basin of water next to the vendor. This same basin would also be used for rinsing bushmeat carcasses, knives, and other tools. Soap was not observed being used in the markets.

“During the day it was only me that washed my hands because I don’t really like having blood on my hands every time I do this job…. I put a small pail beside me in order to wash my hands every time I sell to a client. I don’t like to have hands tainted by blood because I have a large clientele, so I have to avoid that clients coming and finding me with blood on my hands.” *(Bushmeat Vendor*, *Male*, *Marché de la Liberté)*

#### Taste preference for bushmeat drives hunting, procurement and selling practices

Meat taste is important to clientele, and practices have been adopted by all parties in the bushmeat value chain to make sure meat tastes fresh and delicious. These include wiping the butchering knife with only a cloth, washing it with only water (and not soap), being selective about which middle men they work with whose source of meat comes from regions known to have good quality bushmeat, and choosing to only buy animals killed by gun (versus caught in a trap or found dead in the forest), with a visible bullet hole in the carcass. It was common for subjects to say that soap affected the taste of bushmeat, so soap is purposefully not used to wash knives. Other ways of cleaning knives include wiping them in the sand or dirt and heating the knife blade over the fire in the morning before beginning work. Subjects do not take their knives home for fear of being mistaken for bandits, so knives are left at the market nightly. Not all vendors adopt all of these measures, and the more capital a vendor has, the more they can afford to be selective with their bushmeat choices. General consensus is that bushmeat taste is preferred to domestic animal meat because it is “natural,” “fresher” and “not interfered with by humans.” Eating bushmeat is a deeply rooted tradition, with vendors describing ways of butchering specific animals or preparing bushmeat that have been practiced for generations in their home regions, seeing that many of them did not grow up in Kinshasa. Similarly, subjects also report preferring meat caught by rifle, versus by trap, stating that it is fresher, since the animal continues to bleed from the gunshot wound, rather than sitting dead in a trap until the hunter can retrieve it.

#### Ports in Kinshasa are important entry points for both legally and illegally hunted bushmeat

Individuals interviewed at Kinshasa ports, which are gateways for animals arriving by boat from the Équateur and Mai Ndombe provinces, state that the killing and transporting of protected species is still practiced. Those who mentioned Mai Ndombe as a source of bushmeat state there are controls and inspections at the port, so you need to carry more money and pay a bribe to avoid legal repercussions: “This is why you need to make an arrangement (with the inspectors).” After this arrangement is made, vendors are bothered less in the markets about whether they are selling protected species or not, because these animals are often cut up into pieces and unrecognizable, and the markets themselves are not heavily patrolled.

#### Ebola is an illness with mystical origins with short-term market trade impacts

When asked by interviewers, most participants 96.6% (N = 57) interviewed had heard about Ebola, and 72.8% (N = 43) had heard specifically that outbreaks were caused by monkeys. When individuals were asked whether they believed Ebola was real and where it came from, the same detailed story about the mystic origins of Ebola was reported by 50.8% (N = 30) of individuals interviewed. Ebola, according to participants, was caused by a spell placed on a wild animal caught in a trap. After being caught, and before the hunter could return to retrieve the animal, the meat was stolen by a thief. Then a curse was placed on the meat by the original hunter, and the thief, everyone in his/her family, and others in contact with these people became ill and died, including the whole village in some cases. Vendors reported that they tended not to believe the media, or the government, although many of them heard about Ebola on the radio or television, citing a long-standing mistrust of formal institutions. Vendors who did not report this story said they either “don’t know if Ebola is real”, did not believe it existed because they “continued to eat bushmeat and do not die”, or thought “it’s a plot by the government/doctors to get money.”

“During this period [Ebola outbreaks], selling became difficult and yet this story of Ebola is false. What we know is that there was a hunter that caught his game in the forest and some people stole it and ate it and for revenge he put a spell on it and it killed all the people that ate it. But this wasn’t Ebola because if that was the case, we wouldn’t have been selling (at all)….it wasn’t Ebola, it was a spell cast on stolen game.” *(Bushmeat vendor*, *Female*, *Marché de la Liberté)*.

Sales of bushmeat were reportedly negatively affected for all vendors during the Ebola outbreaks, for approximately 2–3 months according to our interviewees. Vendors reported a decrease in the volume of sales, explaining that many customers were scared to purchase bushmeat, particularly because bushmeat was technically banned (although enforcement of the ban was inconsistent). Vendors reported losses in profit during this time, as well as a decrease in supply, saying transporters no longer brought meat, or meat was no longer sent to Kinshasa. When the meat did not sell, vendors would either take the meat home themselves to eat or throw it out into the river, since the supply was sometimes greater than they could consume themselves. Monkey was the species implicated in Ebola outbreaks and vendors reported having difficulty selling monkeys specifically; however, some vendors simply said all bushmeat sales were affected during these times.

#### Bushmeat quantities have declined over time, along with the profitability of the bushmeat trade, but bushmeat remains an important source of income for Congolese in our study

Participants reported that the bushmeat trade has become increasingly difficult, with waning profits, less abundant bushmeat, lower quality and less species diversity. Vendors who had been working in the bushmeat trade for over eight years (on average) tended to mention these issues more than those with less time spent in the bushmeat trade. For instance, vendors reported that now, because hunters have to go so deep into the forest to find animals, meat procurers that go to regions where meat is available rarely report buying directly from hunters. Rather, hunters stay in the forest for long periods of time, and intermediaries carry the meat out of the forest to local villages for sale. As a result, the bushmeat value chain becomes a four-person chain, from hunter to local transporter/seller, to intermediary, who then brings meat to the capital, to vendor, who sells it to the urban consumer. As described by one vendor,

“…we no longer have game [from Mai Ndombe] …Game came in large quantities before [from Bateke], but I don’t know if its sorcery or what …. I don’t know if there was an evil spell cast, but there are no more animals in the forest. Q: An epidemic? A: Yes an illness, a spell that was cast in the forest…. And I don’t know [how long] but its lasted… from 1997, during President Mobutu’s period.” *(Bushmeat Vendor*, *Male*, *Marché d’Ndolo)*

Participants also report longer periods of time for bushmeat procurement than previously, and noted that in the dry season, meat by boat does not arrive as quickly from the Congo River, due to lower water levels.

## Discussion

Our study provides a socio-economic, qualitative analysis of the bushmeat value chain, as well as behavioral insights from actors in multiple markets in Kinshasa. Perceived, actual, and observed occupational risks for actors and their beliefs about wild animals and disease transmission are also presented. We use these findings to highlight areas of interest in the Kinshasa wild market value chain analysis for further investigation. Furthermore, we discuss how beliefs and practices of actors that work with bushmeat might inform risk reduction measures in Kinshasa markets and beyond, as well as the limitations of current work.

### Occupational risk perception and practices

Our study detailed several behavioral practices in bushmeat market vendors that would be considered “risky” for facilitating the spread of zoonotic disease, in that these practices raise the chances of blood-to-blood contact and microbial contamination between wild animals and humans [29, 30]. They include butchering wild animals, frequent cuts while butchering (and hence contact with fresh animal blood of wild animals), not wiping knives (cross contamination of blood between different species and with humans), butchering on surfaces with questionable hygienic practices (microbial contamination) and handling and butchering raw meat with no personal protective equipment (no barrier protection from body fluids). The absence of associating “risk” to these activities by participants versus scientific consensus has been shown in other studies [31]. The question still remains how to frame educational messages to change perception of risk, or to facilitate acceptance of risk reduction strategies in these settings in a low cost-manner and what strategies would be most accepted by this population. While messages during an outbreak event can be effective for a time in reducing bushmeat consumption, inevitably the crisis and urgency fades and people resume their normal buying and selling practices [31]. Messages could be delivered consistently (during non-outbreak times) and focus on human health and reducing human-to-human transmission during outbreaks rather portraying that bushmeat is bad or contaminated. This is not to say educational messages should completely ignore the wildlife-human connection: for example in Eastern Democratic Republic of Congo, while the origins of the 2019 Ebola outbreak were similarly based in mythology initally, the majority of participants also demonstrated knowledge and acceptance that Ebola originated from wild animals and was zoonotic in nature [32]. How to balance messages about risk in populations whose income relies on the very activity you are labeling as “risky” remains a ongoing challenge for social and behavioral science within the One Health framework.

Furthermore, within the bushmeat value chain as described by our participants, actors have clear well-defined roles, which have different hazards. Therefore, messaging, while important to the target population, should also consider differences in risk within these sub-populations too, and is an area for further investigation. For example, survey data in Sierra Leonne with hunters and traders found that traders cut themselves more frequently than hunters, and women more than men [33]. They concluded that workers who butcher, trade and sell bushmeat may be at disproportionate risk versus hunters. Additionally, since these roles were filled mostly by women in other studies [34, 35], women may also be at disproportionate risk. In our study, women were not over-represented as vendors or traders, but vendors/butchers did report frequent cuts during their work at the markets in our study. However, when discussed, most interview participants did not consider butchering “risky” and developed butchering techniques over time to reduce their chances of cutting themselves. Since some association of risk was attributed by participants to butchering, education strategies could focus on covering cuts, and avoiding blood-blood contact whenever possible. It is worth exploring if a butchering mentorship program, in which older bushmeat vendors share learned knife and cutting techniques with younger vendors within a market in an attempt to reduce the frequency of cuts.

Regardless of their role, PPE seems to be universally unaccepted by this population, and also unsustainable should they be made more readily available; given that most participants find them cumbersome, slippery and burdensome, they would not likely prioritize the purchase of protective gear themselves, nor use them should they be provided. Participants that butcher could instead be encouraged to have separate work clothes and shoes that are left at the market to discourage carrying animal blood and other body fluids home. Of course, easy access to clean water and educational campaigns could facilitate more frequent hand washing, but the logistical, infrastructure and political dimensions of installing water in bushmeat markets, managed and funded in different ways, and some of which are informal and along roadsides, makes this option challenging in practice.

### Ebola and health messaging

Our data collection covers a span of three years, from 2016–2018, a time where there were multiple zoonotic outbreak events throughout DRC, and the largest Ebola outbreak in history was occurring in West Africa; an epidemic that was declared over by the WHO in June, 2016 [36]. Participants were all asked about Ebola outbreaks, their origins, and their effect on market supply, demand and price. During these conversations, market participants conveyed a mistrust of the government and institutional messaging, relying instead on word of mouth to inform them about the illness. Participants also reported hearing health messages on the radio and television from the Ministry of Health. However, these messages were largely ignored. The universal mistrust of formal institutions and the rejection of government health messages within the bushmeat sector is important in that it indicates that health messages from these sources will present an uptake challenge for risk mitigation strategies implemented in this population. Such challenges in the face of Ebola have been reported previously in the general population, especially during the outbreak in West Africa from 2014–2016 [37–40].

Bushmeat vendors reported suffering difficult economic times during Ebola, and reported people stayed away from purchasing bushmeat, specifically monkey. They themselves often continued to eat bushmeat, and consumers slowly resumed their buying practices as the outbreak subsided. This could indicate that educational messages aimed at, and successful for a short period of time in preventing bushmeat consumption in the general population are not successful for those in the bushmeat value chain themselves. These findings suggest that the best way to reach at risk populations regarding public health information might be through a combination of formal and informal channels. Since our team needed to obtain local authorization from the head of each market before performing market visits, mobilizing these important actors for dissemination of health information could be strategically beneficial. Radio messages involving popular local shows or radio personalities might also be an effective means of reaching the urban population, especially those in poorer areas without access to television. In Équateur province, participants working in markets did report hearing outbreak education at churches and through word-of-mouth [32], demonstrating the importance of finding creative informal channel for which to disseminate information.

The fact that Ebola was attributed to mythical origins by bushmeat vendors echoes a similar pattern seen during the 2014–2016 Ebola outbreak in Liberia, Sierra Leone and Guinea, when witchcraft was attributed to the origins of the disease [41]. In such instances, communicating risk information when misinformation, stigma, and skepticism were prevalent is challenging [42]. Care must be taken to consider that when the next Ebola outbreak strikes in DRC that many of the most high-risk subjects harbour these same beliefs and that conventional public health messages about Ebola might not be effective for this group. Without proper consideration, the bushmeat trade could go underground and risks associated with the bushmeat trade will not be reduced. Although among a small sample size, churches were found to be a source of educational messaging during two Ebola outbreak events in 2016 and 2018 in Mbandaka after the two Ebola outbreak events in 2016 and 2018 [40] and could be engaged in targeted city quarters with bushmeat markets for dissemination of educational messages around disease prevention for the community that could also reach high-risk populations such as bushmeat vendors.

### Bushmeat movement

The volume of bushmeat in circulation across Central Africa is vast, with consumption of bushmeat in the Congo basin estimated at 1 million metric tons [43] and four times that in a study three years later [44]. There are no estimates for the volume of bushmeat in Kinshasa markets, but the network of markets and city mapping [22] suggests a large volume is traded and consumed annually. The bushmeat value chain to Kinshasa reported by participants in our study indicates three regions in DRC where bushmeat is procured, where meat passes from protected and non-protected forest regions to rural villages, mid-sized towns and even some larger towns before it arrives at the capital for sale. Unsurprisingly, given the Kinshasa City population, and the extensiveness of bushmeat markets throughout the city, this network is both vast and complex. While our analysis of the bushmeat value chain was qualitative and descriptive in nature, it highlights the huge challenges facing a One Health approach in determining and implementing recommendations for improving safety, regulating trade, improving conservation and reducing risk of wild animal contact among the actors. While some animal value chains, particularly those linked to domestic animal production, have defined control points, from animal processing facilities to domestic farms [45], large parts of the wild animal value chain often operate outside traditional regulation and formal facilities [46, 47] and would require a multiactor approach, including government and regulators. Similar conclusions have been reached in other wild animal commodity chain analyses [48], though these remain limited in Central Africa. However, our interviewees named three key provinces where bushmeat is sourced for the Kinshasa. This tells us that coordination among local authorities within DRC, touching on all actors, from regulatory, to conservation, to safety would be required to successfully manage, a huge undertaking. While the task would be vast, a coordinated effort among local authorities should not be wholly discounted. Activities to reduce risk and address environmental issues, such as safe and sustainable hunting practices, consistent enforcement of protect species status and appropriate cold storage for meat could improve the overall human risk and safety involved in the wild animal trade. Aiming for a bushmeat ban, for example, especially during epidemics, can be counterproductive [49–51] and vendors in our study mentioned bribing officials as a means to bypass bushmeat restrictions. A One Health Approach, ensuring consistency in regulations, messaging and more standardized approaches must engage actors, stakeholders and policy makers at multiple levels. This could reduce the chances of spillover or zoonotic outbreaks occurring in a dense urban area where it may more easily spread among humans.

### Sustainability

Reports of bushmeat quality, quantity and species declining over time were not inspired from a question asked directly by interviewers, nor contained in the interview guide. Ecological studies have shown that one of the best predictors of decline is indicated by a decline in average body size of prey [52]. This has been observed in DRC quantitatively via market counts over time [53] and might be explained by deforestation, over-hunting and urbanization. It was true also in IMPACT study data, while limited, that the majority of bushmeat observed and counted was small in body mass (S1–S4 Tables), even in the primate order, though the link to regional ecological decline from data such as this alone is unlikely. However, this is the first evidence of self-reported and un-solicited interview data detailing these findings from market vendors themselves in a qualitative context. Further exploration over time, both in a behavioral, quantitative and ecological context in Kinshasa could shed more details on this decline and offer further insights on the sustainability of the bushmeat value chain long-term. Longitudinal evaluation of these findings might also yield further insights on the necessity of reducing population dependence on bushmeat and hence, reducing risk of zoonotic pathogen transmission between animals and humans.

### Limitations

In our study, participation was limited to subjects working in the animal value chain at markets in Kinshasa and is therefore not representative of the general population, or the consumer population of the market. Bushmeat can be a sensitive subject; some species are protected; attention has been focused on the bushmeat trade and bans and enforcement have targeted bushmeat markets in the context of a disease outbreak. Therefore, there is risk that participants may not be completely forthcoming, their responses may be biased towards things they think the researcher is looking to hear, or their responses are inadvertently crafted to protect their livelihood. This was clear in that sometimes interviews were cut short because participants were suspicious (N = 3) and were not included in the final interview count or acted visibly uncomfortable according to interview notes. In order to build trust with participants and overcome these limitations we used the same two local researchers for the entire study period who had been working in the selected bushmeat markets for many years (PREDICT 1) and had established relationships with market chiefs and market vendors. While we aimed to recruit all participants at a given market, refusals did occur, principally related to the requirement of being audiotaped. However, miscommunication in early data collection means an accurate count of refusals was not obtained.

Kinshasa has not been the site of any major spillover events or outbreaks and species found in the two markets surveyed are not commonly hosts of major diseases such as Monkeypox and Ebola, with the exception of some monkey species. While Ebola questions were consistently asked by interviewers, the lack of an Ebola outbreak in Kinshasa might also result in less perceived risk versus another urban area like Mbandaka, for example, a city in Equateur province which has experienced several Ebola outbreaks. Furthermore, sampling for the IMPACT study was done mostly on weekdays, which meant weekends were underrepresented in the data. There is a chance more participants entered the markets on weekends, when the formal work week was over, or the activity was different, which was not captured in our data.

## Supporting information

S1 Fig PREDICT semi-structured interview guide. The guide was divided into five domains and participants were asked questions from all five domains, though not necessarily the same questions for each interview. Interviewers focused on the following three domains: 3) biosecurity in human environments 4) Illness, medical care/treatment and death of humans 5) Human-animal contact. Participants were required to have indirect contact, defined as animals living in, or entering dwellings, buildings or gardens/crops (e.g. bats roots in roofs) or direct contact, defined as raising, hunting, selling trading or purchasing live, freshly killed or smoked animals.
(DOCX)

S2 Fig PREDICT focus group guide. The guide was divided into three sections and researchers covered all three domains with participants in each focus group. The sections were 1) Contact and context 2) Illness in animals and humans, and 3) Rules and restrictions. As with semi-structured interviews, researchers were allowed flexibility to go with the flow of conversation raised in the group and did not necessarily address all questions in each focus group.
(DOCX)

S3 Fig PREDICT qualitative codebook. The PREDICT qualitative codebook was developed with the flexibility to insert and delete codes as needed during the analysis. The final codebook used for analysis of this manuscript is included.
(XLSX)

S1 Table Ndolo market species count. Each species is indicated by both scientific and common name, unless unidentifiable by taxa. The number of each per day of market observation (either whole or partial animal) is included as well as how the meat is being sold (live, fresh, frozen, smoked).the meat is being sold. Average animal weight and IUCN red list status is also included wherever possible.
(XLSX)

S2 Table Ndolo market pricing. Due to the large diversity sold at the market, prices included are based on scientific name, where possible. Unidentified species prices are also included where possible. Prices are all in USD, with conversion rate (based on rates at approximate date of data collection) are included below.
(XLSX)

S3 Table Gambela market species count. Each species is indicated by both scientific and common name, unless unidentifiable by taxa. The number of each per day of market observation (either whole or partial animal) is included as well as how the meat is being sold (live, fresh, frozen, smoked). Average animal weight and IUCN red list status is also included wherever possible.
(XLSX)

S4 Table Gambela market pricing. Due to smaller diversity of species, pricing is group by taxa only. Prices are all in USD, with conversion rate (based on rates at approximate date of data collection) are included below.
(XLSX)
