# Supplementary material for: Risk perceptions and behaviors of actors in the wild animal value chain in Kinshasa, Democratic Republic of Congo
Source: PLoS One. 2022 Feb 16;17(2):e0261601. doi: 10.1371/journal.pone.0261601 (PMC8849473; doi:10.1371/journal.pone.0261601)
Supplement: S1 Fig — The guide was divided into five domains and participants were asked questions from all five domains, though not necessarily the same questions for each interview. Interviewers focused on the following three domains: 3) biosecurity in human environments 4) Illness, medical care/treatment and death of humans 5) Human-animal contact. Participants were required to have indirect contact, defined as animals living in, or entering dwellings, buildings or gardens/crops (e.g. bats roots in roofs) or direct contact, defined as raising, hunting, selling trading or purchasing live, freshly killed or smoked animals. (DOCX) [file pone.0261601.s001.docx]

**PREDICT-2 ETHNOGRAPHIC INTERVIEW GUIDE**

**GENERAL GUIDANCE**

- This interview guide was designed to be a semi-structured tool to help guide the conversation. In preparation for your interview, tailor the questions to your specific interface, paying particular note to your taxa of interest.
- Similarly, many of the questions are worded in reference to the home. During the interview, ask the questions in reference to both the home and work environments, as applicable.
- In addition, because it is semi-structured, not all questions need to be asked, but please use probes relevant to risk mitigation interventions from Core Themes 3 – 5 and make efforts to ask the final questions on the guide (5e. Perceptions and Knowledge).

**CORE THEMES**

1. Human movement
2. Socioeconomics
3. Biosecurity in human environments
4. Illness, medical care/treatment and death of humans
5. Human-animal contact
6. **HUMAN MOVEMENT**

***GOAL: To understand living environment and ‘home range’ (e.g., how far people travel and why)***

- 1. ***Home***
     1. Where do you live/what kind of dwelling? How many people are in the household? How many rooms? How many are children? Is everyone related? Sleeping arrangements?
     2. How often do you move, if at all? Any seasonality of movements?—e.g., for work, for food, for safety (e.g., against flood, drought, conflict)?
     3. What are the things you do to protect your home (e.g., against predators, animals, outsiders, bad weather)?
  2. ***Work***
     1. What kind of work or activities do you do? What do other household members do? Where do these activities happen?
     2. How do you protect your activities and business interests (e.g., grazing or crop land, business competition, hunting territory, animal stock)?
  3. ***Travel***
     1. How far do you or your household members travel from home, how often? (Follow up on animal related issues: shopping, selling/buying/trading, hunting, transport, etc.) How about in the last year? Please describe where you travel for work.
     2. How do they/you travel (by foot, bike, cart, truck, plane)? Is it ever for overnight? Where stay?
     3. Why are they/you traveling? (work/migrant, family, religion, holidays, to sell/trade/buy animals)
     4. Other family members in other areas of the country? Visit often?
  4. ***Observed environment***
     1. Have there been any changes in the environment: new roads, more boats or ports, fields, buildings, population movement (in or out), land clearing or abandonment, new houses, other new buildings? More or less domestic or wild animals in the area? – types
     2. Who is responsible for the changes? How did these changes come about? Are the changes good or bad?

1. **SOCIOECONOMICS**

***GOAL: To understand a typical day and how money and social standing impact opportunity and risk***

- 1. ***Daily routine***
     1. Tell me about your daily routine (get description of work on a usual day, include purchasing and preparing food, timing of types of meals, responsibilities/duties related to animals, any changes by season)
     2. How do people in the household contribute to earning money and getting food (and water)? If your family has a business related to animals, how often do you help with this? What is your role in helping?
     3. Where do the children play? Who takes care of the children when you are at work?
  2. ***Animal responsibilities***
     1. Describe the animal related jobs and responsibilities for people at every age (i.e., young children, older children, young adults, adults, elderly). What kinds of animals (domestic and/or wild, and types)? Are there differences in responsibilities based on animal type?
     2. What are the skills/knowledge people need to know for each level of animal-related responsibility? (e.g., assisting with feeding vs. birthing, etc.) How is this information learned? To what extent do you think there are risks related to your responsibilities? What skills or knowledge could help prevent risks related to your responsibilities?
     3. Are there differences in responsibilities between boys and girls, men and women, by ethnicity or class?
  3. ***Education***
     1. How many children are currently in school? Until what age do your children go to school? (boys and girls?)
     2. What is your level of education? Why did you stop? Will you plan to complete/continue your study? And why?
  4. ***Economics***
     1. Do you make more money than other people who do the same things as you? Why do you think that is?
     2. Are there times of year when you make less money? What happens then? (e.g., do they find additional work? Is it animal-related?)
     3. Are there times when food is more expensive than others? Tell me about that (e.g., different food availability, seasonal, festival related). If there are times when food is more expensive, are there things that you do to adjust to these periods? Please tell me about that.
     4. Do you think you and your household are better off than most people? Could you do things to make it better?

1. **BIOSECURITY IN HUMAN ENVIRONMENTS**

***GOAL: To determine if any sanitation or hygiene factors could play a role in disease spillover***

- 1. ***Water and food***
     1. Is there a central source of water? What is the source? (e.g., pond, uncovered well, rainwater, taps, covered well) Is there a water source you like better, why?
     2. How far away is the water source? Do animals drink from the same source? Seen at the same water source (e.g., playing, bathing)?
     3. Do you do anything to your drinking water to clean it before you drink it?
     4. How do you store your food (e.g., open containers, covered, hanging, refrigerate, stored in oil)?
     5. Do you eat or drink things where you suspect animal contact (e.g., teeth/scratch marks, feces or urine seen)? What kinds of animals come in contact with your food or water? Is anything done to stop an animal if you see it there? If an animal has contact with your food or water, is anything done before it is consumed by people? Do you regularly clean your food prep station/kitchen and tools? How? How do you usually prepare meat for meals (e.g., uncooked (raw meat or raw blood), blackened, etc.)? Are there times when you prepare meats in ways other than those that you mentioned?
  2. ***Sanitation***
     1. Are there toilets, latrines or other designated areas for human waste? Are these cleaned and used regularly? Are butchering and slaughtering areas separate? To what extent does waste from butchering and slaughtering go to the same place where human waste goes? How often are they cleaned and how? Who does the cleaning? How common is it for the person handling the waste to wash their hands before going to public areas? To what extent is human or animal waste used as fertilizer, fuel, food, etc.?
     2. Are there any official rules or laws about human waste and garbage disposal? What policies/strategies might you suggest that would work better than what is in place now?
     3. Are there any animal pest control laws? To what extent are they effective? Would you change them? How? What do you do to control animal pests?
  3. ***Hygiene***
     1. At home, when are the best times to wash your hands? When do you use soap? How much does soap cost and where get it? In your opinion, what are the most important times to wash up? After doing what activities? (Ask these questions specific to their work environment as well.)
     2. How often and where do you and your household members bathe?

1. **ILLNESS, MEDICAL CARE/TREATMENT, DEATH**

***GOAL: To identify any unusual disease experiences—signs, symptoms and sources***

- 1. ***Household illness***
     1. Is anyone sick right now? Why/how do you think that **name person** got sick? Describe what may have caused their illness. In your opinion, do you think your family members often get sick because of these causes? Has anyone been sick recently?
     2. What do you do when someone in the household gets sick? Who takes care of that person? (e.g., household members, neighbors, children, etc.)
     3. The last time someone was seriously sick what happened (explore when, with what, how did they get sick, who told/consulted, anyone else get sick after, final outcome, any idea of what causes the illness, any precautions taken to prevent further illness, or illness again)?
     4. Has anyone ever had a sickness that people don’t usually get? What happened? Where did it come from? Why do you think this person got sick while others did not?
  2. ***Illness from animals***
     1. Do you know anyone who has gotten sick from an animal in your community in the last year? What animal? What did they get? What happened? Do you know any other diseases/illnesses people can get from animals? How does the animal give the illness to the person? How often does it happen? What is done to prevent illness? What might be done differently to prevent illness?
  3. ***Medical care/treatment***
     1. How sick would you have to feel to stay home and not do your normal routine?
     2. What do you do when you get sick? Where do you go when you are sick?
     3. What are your preferences in terms of traditional medicine, western medicine, or a combination? Please describe any details that affect why you might choose one over the other. How do their treatments differ?
     4. How sick would you have to feel to go to doctor/clinic/hospital? What does that cost? (in time, lost wages/business, transport costs, etc.) How far away?
  4. ***Death***
     1. What is the tradition when someone dies? (Explore if reported to authorities, differ by age or gender, what happens to the body, does the community come together or is it private.) Where and how is the body stored before burial? If someone dies, to what extent is the spread of diseases of concern during burial ceremonies?

1. **HUMAN ANIMAL CONTACT**

***GOAL: To gain knowledge about interactions with animals, animal health and animal perceptions and knowledge.*** *Encourage but don’t lead discussion about which animals. Allow respondent to name the animals. If your taxa of interest is not mentioned, follow up by asking specific questions about your taxa of interest.*

- 1. ***Indirect contact***
     1. What kind of meat do people in your household eat? How do you get it/where does it come from? What is furthest away an animal comes from? Is meat eaten for any special purposes or occasions? (if yes, what kinds of meat and for what occasions/purposes?) For what other purposes is the meat used aside from for food, if any?
     2. Is meat dead or alive when you handle it? If dead(/prepared), how can you tell if it’s good/fresh?
     3. If alive, how long are live animals kept before being sold or eaten? How do you get live animals home? How is meat prepared (raw/undercooked)? Is meat prepared in the same place as other activities? (e.g., preparing vegetables, cleaning babies/changing diapers, where other food or drinking water is stored) Is the area cleaned? Between activities?
     4. Do animals come in or near the dwelling? How do you know animals are there? Which animals? Is anything done to prevent it? What do animals do when they come in or near the house? Do they make a mess or leave excrement? How is it cleaned?
  2. ***Direct contact***
     1. Do you or someone in your household handle live animals? In what context? (e.g., ranching/animal husbandry, hunting, wet markets, work, around dwelling/other building, pets) What types of animals do they have contact with? Do you come into contact with pest animals that come into your homes (i.e., bats, rodents etc.)? How do you protect your home and food from these pest animals?
     2. What are the animals that you keep/raise or sell? How many different kinds of animals? How many of each? For how long do you have the animals?
     3. Where do live animals come from? Where is the furthest away an animal comes from?
     4. Who buys/trades for your live animals? Where do the animals go?
     5. Have you or your family members been bitten, scratched or had bleeding after handling an animal? What kind of animal? Did you do anything for the bite or scratch? How do you protect yourself from being bitten or scratched when you are handling live animals? Has this ever happened at work? What happened? To what extent do you think your job duties could pose a health risk to you? Why and how? What types of things, if any, do you think you could do to reduce health risks while at work?
     6. Where are live animals slaughtered? butchered? Do people buy or sell parts? What kinds of animals? (domestic and/or wild, and types) Is the area or the tools cleaned after? Regularly? (Ask these questions relevant to the work place; as applicable, probe on turnover rates of animals, contact rates with buyers, constant or different/shifting sources of animal product, etc.)
     7. Do you travel with animals? Explore details of the process, specific routes and encounters (e.g., with other animals, with animal transport supporting industries, such as holding areas, restaurants, hotels) along the way. Explore for differences over time in animal handling, e.g., seasonality, legal, religious, animal reproduction.
  3. ***Animal products/rituals***
     1. Other uses of animals or animal products—e.g., as pets, medicine, magic, fertilizer***,*** for trading. Are there animals, animal parts, or animal products that are used for treatments? What type of treatments? At holiday or holy days? During different seasons? (domestic and/or wild, and types of animals)
     2. Rules for children around wild animals as pets, playing with wild animals or dead animals. At what age do children start hunting/trapping in this community? Both boys and girls?
  4. ***Animal health***
     1. How do you care for your animals: how are they fed, what do they eat, where do they eat/graze and sleep? Are they segregated or all together? Differences by season? day/night? Does anyone live or stay with the animals? Is there a central area for animal waste? How often are animal cages, stalls, or penned areas cleaned? Who cleans them? (domestic and/or wild, and types of animals)
     2. Do the animals get veterinary care? Vaccinations?
     3. How do you know when an animal is sick? What’s the first thing you do about a sick animal? Do illnesses vary by animal type?
     4. Have you seen an animal outbreak or die-off in your community in the last year? What happened? What caused it? What types of things were done to deal with it?
  5. ***Perceptions and knowledge***
     1. What are the most unusual, rare, specialty, or exotic animals anyone can buy?—seasonal? Expensive? Who buys them?
     2. Are there any animals you avoid eating? Why? Ever heard of anyone eating/selling dead or infected animals? What types of animals? Do people ever eat non-domesticated animals/wildlife? Where do they get them?
     3. Who usually buys wildlife products? From what animals? Have there been changes over time?
     4. What do you do when you find a dead animal? What types of animals have you found dead?
     5. What laws or health recommendations about animals do you know (e.g., limiting/outlawing hunting, reporting and culling of sick animals; probe as relevant to your interface, e.g., animal rearing, meat preparation, meat markets, etc.)? Are there policies about hunting? about animal markets? How do most people learn about these policies? Do you think they are a good idea? Why or why not? Do you think that they are helping? Why or why not? How do you think they could be improved? What do you think are the biggest barriers to preventing the spread of disease from animals to humans? What types of things have been most helpful in preventing the spread of disease from animals to humans? If you could do three things to help prevent the spread of disease from animals to humans in your community, what would you do?
