# Supplementary material for: Risk perceptions and behaviors of actors in the wild animal value chain in Kinshasa, Democratic Republic of Congo
Source: PLoS One. 2022 Feb 16;17(2):e0261601. doi: 10.1371/journal.pone.0261601 (PMC8849473; doi:10.1371/journal.pone.0261601)
Supplement: S2 Fig — The guide was divided into three sections and researchers covered all three domains with participants in each focus group. The sections were 1) Contact and context 2) Illness in animals and humans, and 3) Rules and restrictions. As with semi-structured interviews, researchers were allowed flexibility to go with the flow of conversation raised in the group and did not necessarily address all questions in each focus group. (DOCX) [file pone.0261601.s002.docx]

**PREDICT-2 FOCUS GROUP GUIDE Version 3, August 1, 2017**

**GENERAL GUIDANCE**

- This focus group guide was designed to be a semi-structured tool to help guide the conversation. In preparation for your focus group, tailor the questions to your specific interface, paying particular note to your taxa of interest.
- Similarly, many of the questions are worded in reference to the home. During the interview, ask the questions in reference to both the home and work environments, as applicable.
- In addition, because it is semi-structured, not all questions need to be asked, but please make efforts to use probes relevant to risk mitigation interventions.

**MAPPING ACTIVITY**

The focus group discussion is initiated by naming all of the animals that can be found in the community. The goal of this exercise is to explore animal diversity.

The community mapping activity locates where the different kinds of animals can be found relative to the site of the focus group. It should be emphasized that this will not be an ‘accurate’ map. This exercise is designed to assess the distribution and overlap of animals. Prompts such as ‘anywhere else?’ should be used. The animal list will contain insects, reptiles and fish. ***Map only mammalian and avian species***.

These two activities together should be limited to 10-15 minutes. The themes to be explored in the discussion are 1) contact and context, 2) illness in animals and humans, and 3) rules and restrictions. Events such as animal die-offs should be added to the map, if they are discussed.

1. **Contact and context**
2. Which of these animals do you see the most often? The least? (Probe: where, why)
3. What animals do you come into physical contact with? (Probe: where, why, how often)
4. Which of these animals do you eat?
   - Where do you get them? What is furthest away an animal comes from?
   - How are they prepared? Which are for special occasions only?
   - Where are live animals slaughtered? butchered? Do people buy or sell parts? What kinds of animals? (domestic and/or wild, and types) Is the area or the tools cleaned after? Regularly? (Ask these questions relevant to the work place; as applicable, probe on turnover rates of animals, contact rates with buyers, constant or different/shifting sources of animal product, etc.)
5. What are animals good for other than food? (probe: labor, medicinal, magic, pets, by-product uses)
   - Are there animals, animal parts, or animal products that are used for treatments? What type of treatments? At holiday or holy days? During different seasons? (domestic and/or wild, and types of animals)
   - What are the most unusual, rare, specialty, or exotic animals anyone can buy?—Are they seasonal? Expensive? Who buys them? Who usually buys wildlife products? From what animals? Have there been changes over time?
6. Which animals come into buildings or places where people are? Is water shared with animals?
   - What kinds of animals come in contact with your food or water? Is anything done to stop an animal if you see it there? If an animal has contact with your food or water, is anything done before it is consumed by people?
7. What types of animals do you have contact with? Do you come into contact with pest animals that come into your homes (i.e., bats, rodents etc.) (probe: which animals, all methods used)? How do you protect your home and food from these pest animals?
   - Who takes care of the animals? (Probe: who, specific jobs, animal movements) What kinds of animals (domestic and/or wild, and types)? Are there differences in responsibilities based on animal type?
   - What are the skills/knowledge people need to know for each level of animal-related responsibility? (e.g., assisting with feeding vs. birthing, etc.) How is this information learned? To what extent do you think there are risks related to your responsibilities? What skills or knowledge could help prevent risks related to your responsibilities?
8. **Illness in animals and humans**

*Animals*

1. What happens when animals get really sick? How are animals cared for? Have you seen an animal outbreak or die-off in your community in the last year? What happened? What caused it? What types of things were done to deal with it?
   - How do you know when an animal is sick? What’s the first thing you do about a sick animal? Do illnesses vary by animal type?
2. Has this happened recently? Do people try to hide animal sickness?
   - What caused it? What types of things were done to deal with it?
3. Is animal sickness reported to anyone? (probe for differences between wild and domestic animals)
4. Have any animals been destroyed or killed by authorities? Describe.
5. What do you do when you find a dead animal? What types of animals have you found dead?
6. (probe: eaten, buried, left to rot, depends if wild or not)

*Humans*

1. What is the most unusual or memorable sickness anyone has had? What happened?
2. What are the causes of illness or sickness?
3. What do you do when someone in the household gets sick? Who takes care of that person? (e.g., household members, neighbors, children, etc.)
   - The last time someone was seriously sick what happened (explore when, with what, how did they get sick, who told/consulted, anyone else get sick after, final outcome)? Any idea of what causes the illness? Any precautions taken to prevent further illness, or illness again)?
   - Has anyone ever had a sickness that people don’t usually get? What happened? Where did it come from? Why do you think this person got sick while others did not?
4. Do you know anyone who has gotten sick from an animal in your community in the last year? What animal? What did they get? What happened?
   - Do you know any other diseases/illnesses people can get from animals? How does the animal give the illness to the person? How often does it happen? What is done to prevent illness? What might be done differently to prevent illness?
5. What do you know about animals that can give you infections or diseases?
6. **Rules and restrictions**
7. Are there places in the community where you aren’t allowed to go? Why not?
8. Are there any rules about hunting or trapping animals? (e.g., limiting/outlawing hunting, reporting and culling of sick animals; probe as relevant to your interface, e.g., animal rearing, meat preparation, meat markets, etc.)?
   - Are there policies about hunting? about animal markets? How do most people learn about these policies? Do you think they are a good idea? Why or why not? Do you think that they are helping? Why or why not? How do you think they could be improved?
   - What laws or health recommendations about animals do you know (e.g., limiting/outlawing hunting, reporting and culling of sick animals; (Probe as relevant to your interface, e.g., animal rearing, meat preparation, meat markets, etc.)?
9. Are there any animals that you don’t eat or that are avoided? Why?
   - Have you ever heard of anyone eating/selling dead or infected animals? What types of animals? Why do people eat them? Do people ever eat non-domesticated animals/wildlife? Where do they get them?
10. Are there official rules or laws about garbage disposal? Human waste? Animal waste? What policies/strategies might you suggest that would work better than what is in place now?
11. Is garbage a problem in this community? What’s the problem? What do you think are the biggest barriers to preventing the spread of disease from animals to humans? What types of things have been most helpful in preventing the spread of disease from animals to humans? If you could do three things to help prevent the spread of disease from animals to humans in your community, what would you do?
